# Supplementary material for: Complementary Targeting of Rb Phosphorylation and Growth in Cervical Cancer Cell Cultures and a Xenograft Mouse Model by SHetA2 and Palbociclib
Source: Cancers (Basel). 2020 May 17;12(5):1269. doi: 10.3390/cancers12051269 (PMC7281234; doi:10.3390/cancers12051269)
Supplement: Supplementary file 1 [file cancers-12-01269-s001.pdf]

Supplementary Materials: Complementary Targeting of Rb Phosphorylation and Growth in Cervical Cancer Cell Cultures and a Xenograft Mouse Model by SHetA2 and Palbociclib

Amy L. Kennedy, Rajani Rai, Zitha Redempta Isingizwe, Y. Daniel Zhao, Stanley A. Lightfoot and Doris M. Benbrook

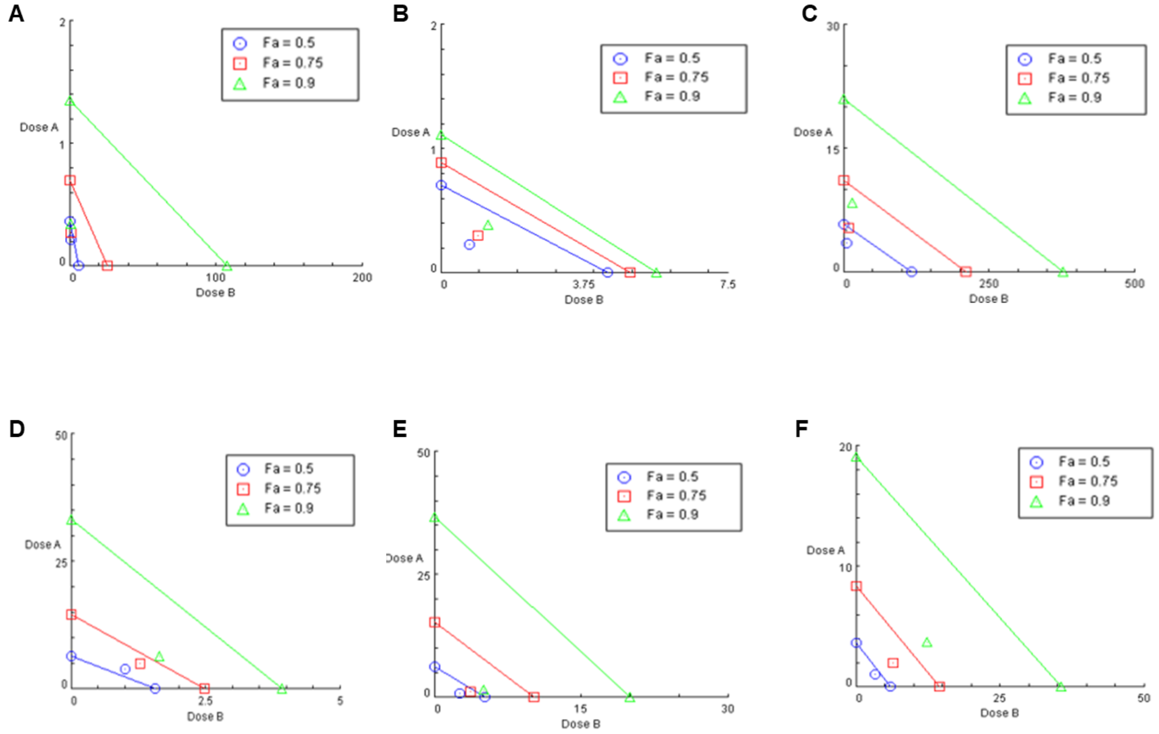

Figure S1. Replicate Isobolograms for (A-B) C-33A, (C-D) CaSki, and (E-F) SiHa cervical cancer cell lines.

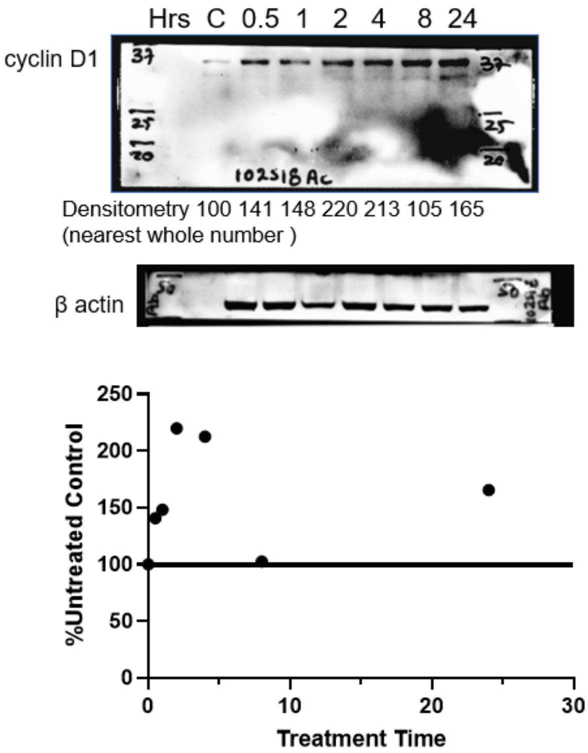

Figure S2. Western blots of SiHa cultures treated with NNK for the indicated amount of time or control solvent for 0.5 h (C) are shown on top. Densitometry of the western blots is shown below. * Membranes were cut to reduce non- specific binding bands.

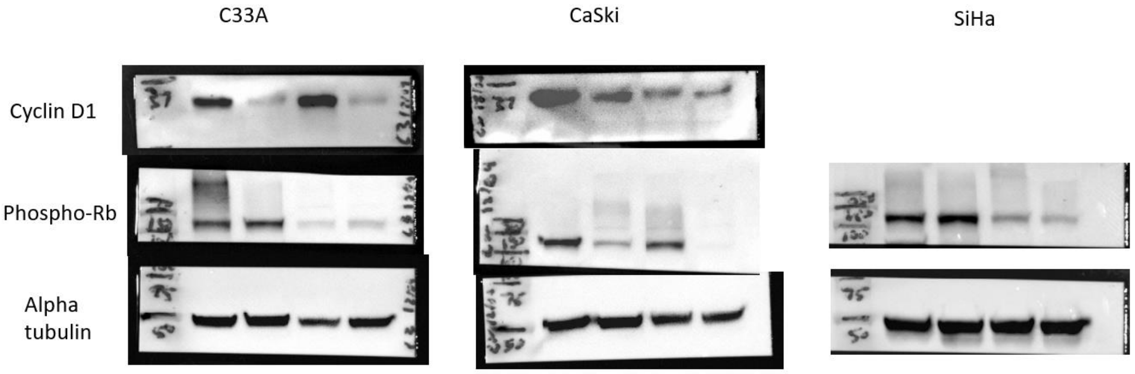

Figure S3. Full Western Blot Images for Figures 2 and 3.
